# Determining freshwater *p*CO_2_ based on geochemical calculation and modelling using PHREEQC

**DOI:** 10.1016/j.mex.2021.101430

**Published:** 2021-06-25

**Authors:** Leonie Pötter, Ralph Tollrian, Frank Wisotzky, Linda C. Weiss

**Affiliations:** a Department of Animal Ecology, Evolution and Biodiversity, Ruhr–University Bochum, NDEF, Universitätsstraße 150, D-44780 Bochum, Germany; b Hydrogeology Department, Ruhr–University Bochum, Universitätsstraße 150, D-44801 Bochum, Germany

**Keywords:** PHREEQC, *p*CO_2_, Freshwater acidification, Total CO_2_ concentration (TCO_2_), Total alkalinity (TA), Total inorganic carbon (TIC), Long–term data, *p*CO_2_–linked–process components, Climate change, Global carbon cycle

## Abstract

Fossil fuel combustion results in rising atmospheric carbon dioxide (CO_2_), which is known to impact the global climate and the oceans. Latest insights indicate that rising atmospheric CO_2_ levels also affect CO_2_ partial pressure (*p*CO_2_) in freshwaters, where *p*CO_2_ is controlled by a multitude of parameters. However, up to date there is no standardized method, which allows the determination of current and past freshwater *p*CO_2_ levels. Ideally methods should incorporate numerous hydrogeochemical and -physical factors to reflect the interplay of all interacting components and their effect on *p*CO_2_. We here describe the application of the geochemical program PHREEQC. This freeware serves as an easy method enabling a plausible and comprehensive analysis of *p*CO_2_ for field, laboratory, and especially long-term data. We present the use of the different input parameters of a laboratory- and a field long-term monitoring dataset including dissociation constants of carbonic acid measured as total inorganic carbon (TIC) and total CO_2_ concentration (TCO_2_) or total alkalinity (TA), together with hydrogeochemical and -physical parameters. Based on current literature and our analyses PHREEQC appears a solid strategy to determine freshwater *p*CO_2_ that can moreover be used for long-term datasets.

• Comprehensive analysis of *p*CO_2_ for field, laboratory, and long-term data.
• PHREEQC is not dependent on just one sampling method or parameter scheme.
• PHREEQC includes testing the plausibility of a water analysis and enables the assessment of the quality of the laboratory analysis, as well as automatic calculation of all relevant aquatic complexes.

## Graphical Abstract

**Figure:**
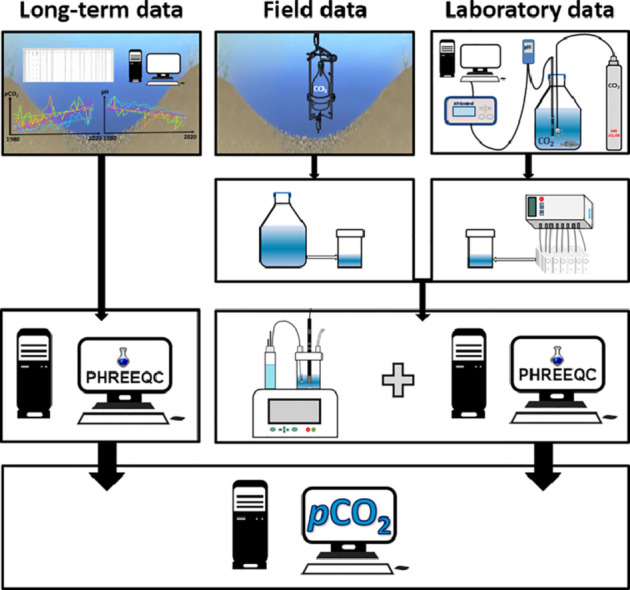

Specifications table

**Table:** 

Subject Area:	Environmental Science
More specific subject area:	*Limnology; Hydrochemistry*
Method name:	*PHREEQC*
Name and reference of original method:	Parkhurst, D. L., and Appelo, C. A. J. (1999). User's Guide To PHREEQC (version 2) – A computer program for speciation, batch-reaction, one-dimensional transport, and inverse geochemical calculations. *US Geol. Surv.Water-Resour. Investigat. Report 312* (pp. 99–4259) Parkhurst, D. L., and Appelo, C. A. J. (2013). Description of Input and Examples for PHREEQC Version 3 – A Computer Program for Speciation, Batch-Reaction, One-Dimensional Transport, and Inverse Geochemical Calculations (p. 497). *US Geol. Surv.Techn. and Meth.*; Book 6, Chapter S43. doi:10.1016/0029-6554(94)90020-5
Resource availability:	https://www.usgs.gov/software/phreeqc-version-3

## Methods details

### ***1. INTRODUCTION/BACKGROUND***

With ongoing anthropogenic activities of fossil fuel combustion, CO_2_ constantly accumulates in the atmosphere, from where it enters the global carbon cycle [10]. It equilibrates with the ocean water, where it chemically reacts and decreases ocean pH; a phenomenon commonly known as ocean acidification [6,^11^,^12^,^14^,^17^,^19^,26]. Similarly, CO_2_ accumulation in inland waters was found to be equal or larger than in the ocean [10]. Thus, inland waters have been discussed as hotspots of biogeochemical activity that also act as carbon sinks [10]. Recently, it has been suggested that increased fossil fuel combustion also affects the carbonate equilibria and *p*CO_2_ levels in freshwater systems, which can even lead to decreasing pH ([10,^22^,^23^,25]; reviewed in [15,29]).

To study this, it is first of all important to chemically describe the freshwater carbonate system. This comprises totally dissolved inorganic carbon content (TIC) that can dissociate into bicarbonate (HCO_3_^–^), carbonate (CO_3_^2–^) and aqueously dissolved CO_2_ (or H_2_CO_3_*) [2]. Aqueously dissolved CO_2_ consist of two pools, i.e. dissolved free CO_2_ and its hydrated form H_2_CO_3_ [9]. The concentration of these system components in combination with the calcium and magnesium concentrations, is influenced by the carbonate's equilibria [2,27]. All these components regulate pH and determine the buffering capacity [2,27], as well as the entire chemical balance in surface waters [9]. Thus, aqueously dissolved CO_2_ is a central variable in aqueous chemistry and is often displayed as CO_2_ partial pressure (*p*CO_2_) in a solution [9]. Factors controlling freshwater *p*CO_2_ are atmospheric *p*CO_2_, the water source (e.g. groundwater, run–off), the residence time of CO_2_ in water, the gas transfer velocity (e.g. changes in precipitation rates, wind patterns, etc.) and the underlying waterbody's geology (e.g. limestone or grey weck, etc.) ([30]; reviewed in [15]). This is further complicated by the surrounding soil respiration rate, terrestrial productivity, and land-use but also by biological processes including heterotrophic and autotrophic activities [3,^4^,^8^,24]. All these factors directly or indirectly affect the aquatic carbon system and are therefore regarded as *p*CO_2_–linked–process components (see Table 1) rendering *p*CO_2_ analyses in freshwater complicated. For a thorough understanding of dynamics and mechanisms regulating freshwater *p*CO_2_ levels and impacts of climate change linked effects, the analysis of past *p*CO_2_ development is pivotal.

**Table 1:** The direct or indirect influence of *p*CO_2_–linked–process components on freshwater *p*CO_2_ levels. *p*CO_2_–linked–process components are regulating and controlling mechanisms and developments of natural processes. Further contributing factors like climatic and geographical regions, agriculture, as well as hydrogeochemical, and biological processes also influence *p*CO_2_ levels. All these parameters and processes are affected by increased atmospheric *p*CO_2_ by which freshwater *p*CO_2_ is discussed to change. The climate regime has a general influence on the different spheres/processes leading to variation in *p*CO_2_ across freshwater systems and the thereby connected impacts on the *p*CO_2_. The given *p*CO_2_–linked–process components can be coded in PHREEQC in the form of keywords or identifiers e.g., the amount of ion species distribution (e.g. Mg^2+^, Ca^2^^+^, HCO_3_^–^, H_2_SO_4_, NO_2_^–^, NH_4_^+^, etc.) and other hydrogeochemical and -physical parameters such as temperature, pH, density, etc.. **Abbreviations:** DOM: dissolved organic matter, DIC: dissolved inorganic carbon; DOC: dissolved organic carbon; POM: particulate organic matter; POC: particulate organic carbon. ‘EQUILIBRIUM_PHASES_REACTION_TEMPERATURE’: PHREEQC keyword calculating gas water exchange reactions of open- or closed systems.

		*p*CO_2_–linked–process
	Hydrogeochemical and biological	components to be reflected in
Climate regime	processes	PHREEQC

Therefore, a precise determination of current and past freshwater *p*CO_2_ levels, as well as associated *p*CO_2_–linked–process components calls for a strategy that permits the analysis of field, laboratory and long-term data. Further, an analysis involving not only inorganic but also organic CO_2_-species is pivotal. The geochemical computer software PHREEQC includes both of these CO_2_-species together with numerous hydrogeochemical and -physical factors for *p*CO_2_ calculation and modelling ([20]; 2013). With PHREEQC a wide range of equilibrium reactions between water and minerals, ion exchangers, surface complexes, solid solutions, and gases can be simulated [5]. This improves the analysis by reflecting the interplay of all interacting components and their effect on the aquatic *p*CO_2_.

We here demonstrate the dual use of PHREEQC as a calculation-tool for laboratory datasets based on dissociation constants of carbonic acid. Further, we describe the application of PHREEQC for a field data e.g., stem from long-term monitoring datasets analyzing.

### **PHREEQC**

PHREEQC version 3 is a computer program written in C and C++ programming and designed to perform a wide variety of aqueous geochemical calculations simulating chemical reactions and transport processes in water [20,21]. It is freely available (e.g. https://www.usgs.gov/software/phreeqc-version-3).

### ***2. METHODOLOGY***

With PHREEQC one can evaluate the quality and plausibility of a water sample based on this ion–balance error (i.e. the percent charge–balance error calculated as (100(cations–anions))/(cations + anions) [2]. With this, the positive and negative charges resulting from the single ion charges is determined [21]. As water is electrically neutral, the sum of the positive charges is approximately equal to the sum of the negative charges. Thereby only analyses of water samples with an ion-balance error ≤5% are discussed to be tolerable [2,31]. If errors exceed 5% either not all ionic compounds were reflected in the analysis, or individual compound concentrations may be over- or underestimated or individual analysis results are incorrect [2].

#### **Calculation of pCO**_**2**_**and associated complexes**

With respect to minerals in a water sample the saturation state (Ω) and the activities of free ions in solution can be calculated [2]. Therefore, the solubility product K is compared to the analogue product of the activities derived from water analyses [2]. Further, saturation conditions or the saturation state (Ω) may also be calculated as the ratio between IAP (ion activity product of the dissociated chemical species in solution) and K (the solubility product of the mineral) [2]:

(1) Ω=IAP/K

There is an equilibrium, when Ω = 1, Ω > 1 indicates a supersaturation and Ω < 1 subsaturation. For larger deviations from the equilibrium, a logarithmic scale can be used for the saturation index (SI) [2,31]:

(2) SI=log(IAP/K)

The SI of different mineral phases, stored in the program's database, is reflected in the PHREEQC output–sheet (see Tab. S7). For dissolved gases the denoted value is given as the common logarithm of partial pressure. Therefore, the calculated *p*CO_2_ is given as the logarithmic value of the saturation index (SI) of CO_2(g)_ [2,31]:

(3) $$pCO_{2}=\;\text{lo}{g^{\text{SI}}}^{\text{CO}2(g)}$$

#### **PHREEQC: input of parameters**

Program keywords and identifiers can be used for the input of certain hydro- as well as chemical and physical parameters such as pH, temperature, ion species next to the dissociation constants of carbonic acid (e.g. TCO_2_ or TA). Parameters obtained from field or long-term data can be entered into the input sheet in following sequence. All given input options are based on Appelo and Postma [2] and Wisotzky et al. [31].

1 To headline the analysis the keyword ‘Title’ is given at the beginning of the input file.
2 Next the keyword ‘SOLUTION’ has to be entered directly under the ‘Title’. Beneath the keyword ‘SOLUTION’, the unit is provided e.g. as ‘mg/l’ (Identifier: ‘units’). Deviating units, e.g. ‘mmol/l’, are written after the corresponding parameter.
3 The parameters temperature, pH, pe and density are provided with the keyword's ‘temp’, ‘pH’, ‘pe’ and ‘density’ (Table 2). Pe stands for the Péclet number (pe = advective transport rate/diffusive transport rate). If density and the pe-value are not available, a default pe (= 4.0) and a density of 1.0 can be used. The pe of 4 is used in the initial equilibrium calculation for all other redox couples, e.g. of H_2_O/O_2_ [2].

**Table 2:** **A)** PHREEQC Input-example in which the parameters can be entered using keywords. Default pe-value (pe = 4) equating to 230 mV at the given temperature. Total CO_2_ given by C (4) as CO2 in mg l^−1^ = (K_S4.3_ + K_B8.2_ (mmol l^−1^) * 44 mg mmol^−1^). The described alternatives for *p*CO_2_ determination via alkalinity or identify of CO_2_ as carbon (without details of the value) are given as Alkalinity (Alkalinity in mg l^−1^ = (K_S4.3_ (mmol l^−1^) *100,089 mg mmol^−1^ (M CaCO_3_)) / 2) # or C as C (C in mg l^−1^= (K_B8.2_ + K_S4.3_ (mmol l^−1^) * 12 mg mmol^−1^) [31]. An example sheet for M4 can be found in the supplementary material (S1). **B)** Example of a model-calculation for the solubility of calcium carbonate and CO_2_ in water.

Parameters	Units	PHREEQC–Keyword	Input–Parameter(to be determined)
		**A)** Title	
		SOLUTION # orSOLUTION_SPREAD	
		pe	
Units of further parameters	mg l^−1^ or mmol l^−1^	units	
Temperature	°C	temp	
pH–value		pH	
Density	mg kg^−1^	density	
total CO_2_ concentration (TCO_2_) C	mg l^−1^ mg l^−1^	C(4) as CO2 # or C as C	
Alkalinity	mg l^−1^	Alkalinity	
		Ca	
		Cl	
		Mg	
		S(6)	
		K	
		Na	
		O(0)	
		Si S S(6) S (-2) N	
		N(+5) N(+3) N (0)	
		P Al Fe(+2) Fe(+3) Mn Mn(+3) Mn(+2) Amm F B Li Br Zn Cd Pb Cu Cu(+2) Cu(+1) END	
		**B)** Title SOLUTION pH temp EQUILIBRIUM_PHASES Calcite CO2(g) END
4 Here it is important to enter the measured distribution of carbon in a complete form of carbonic acid species determining *p*CO_2_ and all CO_2_-components [31]. The values for K_S4.3_ (hydrogen carbonate) and K_B8.2_ (free carbonic acid) determined by titrations give the total concentration of dissolved CO_2_.This measured distribution of the carbonic acid species can be reflected in the input file in the following ways [31]. The calculations relate to the following information:
  (a) It is possible to specify CO_2_ as carbon without valence:
    $$``C\;\text{as}\;C"\left(C\;\text{in}\;\text{mg}\;l^{-1}=\left({K_{B}}_{8.2}+{K_{S}}_{4.3}\left(\text{mmol}\;l^{-1}\right)\right)*12\;\text{mg}\;{\text{mmol}}^{-1}\right)$$
    In this case, the program distinguishes the possible oxidation states in C (4) for CO_2_ and C (-4) for CH_4_ and establishes a redox equilibrium according to the iron specification (reference to the identifier “redox Fe(2)/Fe(3)”) [31].
  (b) Another possibility is to display the titration results as carbon valence:
    $$``C\;\left(4\right)\;\text{as}\;{\text{CO}}_{2}"\left(C\;\left(4\right)\;\text{in}\;\text{mg}\;l^{-1}=\left(K_{B8.2}+\;K_{S4.3}\right)*44\;\text{mg}\;{\text{mmol}}^{-1}\right)$$
    Here, all carbon in the form of carbonic acid species is given as C (4). By specifying the valence with brackets after the element (C (4)), the speciation of the carbon in methane is explicitly excluded, in contrast to a). Further, if the equilibrium pe-value of the iron redox couple is not in the negative range or it prevails only weakly reducing conditions, the program does not calculate methane concentrations [31].
  (c) Alternatively, the hydrogen carbonate determined by titration can be given in the form of alkalinity. In PHREEQC the alkalinity is always stated as CaCO_3_ equivalents. The following stoichiometry reaction is decisive for its determination [31]:
    $$\text{CaC}O_{3}+H_{2}CO_{3}={\text{Ca}}^{2^{+}}+2\text{HC}{O_{3}}^{-}$$

Only half of the hydrogen carbonate measured in the water comes from the calcite solution, the other half from the carbonic acid. Therefore, the equivalent amount of calcite, based on the acid capacity (K_S4.3_), has to be divided by 2 [31]:

$${\text{Alkalinity}\;\text{in}\;\text{mg}\;l}^{-1}=\left(\left(K_{S4.3}\left({\text{mmol}\;l}^{-1}\right)*100,089\;{\text{mg}\;\text{mmo}\;l}^{-1}\left(M\text{CaC}O_{3}\right)\right)/2\right)$$

or

$${\text{Alkalinity}\;\text{in}\;\text{mg}\;l}^{-1}=\left(K_{S4.3}\;{\text{mmol}\;l}^{-1}*0.5\right)*100,089\left({\text{CaCO}}_{3}\;{\text{mg}\;\text{mmo}\;l}^{-1}\right)$$

### ***3. METHOD VALIDATION***

We here describe how *p*CO_2_ and all aquatic complexes can be calculated from a water sample of a laboratory dataset and a long-term monitoring dataset with PHREEQC.

The laboratory dataset is based on the dissociation constants of carbonic acid, which is directly measured as total CO_2_ concentration (TCO_2_) and total alkalinity (TA) via endpoint–titration ((K_S4.3_) for TA, acid and base capacity (K_S4.3_ + K_B8.2_) for TCO_2_). The dissociation constants TCO_2_ and TA are critical input parameters for PHREEQC-analyses as they dependent on water chemistry and buffering capacity of the respective impoundment. In the PHREEQC-input sheet TCO_2_ is represented by the keyword C(4)as CO2 (i.e. TCO_2_) and accordingly as Alkalinity for TA (Table S1; [2,31]).

From a long-term monitoring dataset the annual average of the total inorganic carbon (TIC) can be converted from the total CO_2_ concentration. For both data sets, pH and temperature in combination with a multitude of hydrogeochemical and -physical parameters were included in the *p*CO_2_-analysis to reflect the interplay of many interacting components and their effect on *p*CO_2_ (Table 2; Tables S1; S5; S6). A problem with long-term data is, that the datasets are often limited in the hydrogeochemical parameters they provide. Hence, we here tested how important the multitude of factors is for the analysis. We therefore performed our analyses with and without the multitude of factors to determine possible deviations between calculated *p*CO_2_ (with an ion–balance error ≤5%) compared to modelled *p*CO_2_ (with an ion–balance error ≤99%). In the following, the abbreviations *p*CO_2_[TCO_2_] and *p*CO_2_[TA] stand for PHREEQC-analyses based on total CO_2_ concentration (TCO_2_) or total alkalinity (TA). TCO_2_ measured by endpoint-titration via acid-(K_S4.3_) and base capacity (K_B8.2_) and TA being determined via alkalinity using an endpoint-titration of the acid capacity (K_S4.3_). The symbol (+) stands for PHREEQC–analyses including the parameters pH, TCO_2_ or TA, temperature, as well as detailed hydrogeochemical and -physical parameters and with a calculated output ion–balance error of ≤5% (listed in Tables 3 and 4; Tables S1; S5–S7). The symbol (–) stands for PHREEQC–analyses based on the parameters pH, TCO_2_ or TA, temperature, and density only; neglecting hydrogeo- and chemical parameters as well as a calculated output ion–balance error of ≥99%.

**Table 3:** The concentrations of all elements in the M4 medium of a water sample of the laboratory dataset with modified M4-medium.

Parameters	Units	M4–Final Medium
	g 100 ml^−1^	ml l^−1^
CaCl_2_ 2H_2_O	29.38	1.0
MgSO_4_	24.66	0.5
KCl	5.80	0.1
NaHCO_3_	6.48	1.0
NS2SiO_3_ 9H_2_O	2.50	0.2
NaNO_3_	0.27	0.1
KH_2_PO_4_	0.07	0.1
K_2_HPO_4_	0.18	0.1

**Table 4:** Measured pH and temperature-results ((LL-Aquatrode plus Pt 1000 F/4 mm; Metrohm AG, Herisau, Switzerland), as well as determination-results of acid- (K_S4.3_) and base capacity (K_B8.2_) using endpoint-titration, calculated total CO_2_ concentration and calculated alkalinity of a direct sample of laboratory data.

Results of endpoint–titration	Base capacity(KB _8.2_)	Acid capacity(KS _4.3_)
pH–value	6.449	6.363
mean pH	6.406	
Temperature (°C)	22.3	21.9
mean temperature (°C)	22.10	
m_1_ (g)	184.36	160.19
m_2_ (g)	84.36	60.17
V_2_ (ml)	100.00	100.02
V_1_ (NaOH/HCl in ml)	0.862	0.926
pH value (Endpoint)	8.25	4.28
KB _8.2_ / KS _4.3_	0.862 mmol l^−1^	0.926 mmol l^−1^
total CO_2_ concentration (TCO_2_)	78.664 mg l^−1^	
Alkalinity (TA)	46.341 mg l^−1^	

## **Material and procedures**

### **Laboratory data**

#### Medium

We used a modified version of the artificial M4 medium in all laboratory experiments to ensure a constant water source for the *p*CO_2_ analysis of a water sample (described by the Organization for Economic Cooperation and Development (OECD) in guideline 202 in 2004 [18] ; Table 3). This medium contains *p*CO_2_–linked–process components in the form of the listed ion species (see Table 3; S1).

We freshly prepared 5 L of M4 medium 48 h at the beginning of the experiment, which was split into 4 different experimental conditions of 1 L media. In these we adjusted four different levels of pH (control condition of pH 8.0, *p*CO_2_-conditions with pH 7.0, 6.7 and 6.4 via CO_2_ aeration (Air Liquide-ALIGAL 2)). Elevated *p*CO_2_–conditions were maintained and monitored with computer–controlled pH probes and solenoid valves (AT–control system, AquaMedic GmbH, Germany) with differences in pH not higher than ± 0.05 units. From each condition we sampled 200 mL for endpoint–titration. Each treatment was independently replicated three times (*n* = 12).

#### pH and temperature

Temperature and pH were determined with a pH electrode and an integrated temperature sensor (LL-Aquatrode plus Pt 1000 F/4 mm; Metrohm AG, Herisau, Switzerland). pH was calibrated using three low ionic strength pH buffers (pH 4.00/7.00/9.00 (at 25 °C); Metrohm AG, Herisau, Switzerland-conductivity standard 100 μS/cm-traceable to the NIST (National Institute of Standards and Technology, Gaithersburg, USA)). Measurements were performed directly after calibration of the probes.

#### *Total CO_2_ concentration (TCO_2_) and total alkalinity (TA)*

TCO_2_ of each water sample was determined via endpoint–titration using an automated electro–titration system (Titrino plus 848 combined with LL-Aquatrode plus Pt 1000 (F/4 mm) probe; Metrohm AG, Herisau, Switzerland). We determined acid- (K_S4.3_) and base capacity (K_B8.2_) according to DIN 38409-7:2005-12, German standard methods for the examination of water, waste water and sludge-general measures of effects and substances (group H)-Part 7: Determination of acid and base–neutralizing capacities (H 7) [13]:K_B8.2_ = Base capacity to pH 8.2 with DIN 38409 – H7–4–1K_S4.3_ = Acid capacity to pH 4.3 with DIN 38409 – H7–2     

(4) $$K_{S4.3}=\left[\text{HC}{O_{3}}^{-}\right]+2\left[C{O_{3}}^{2-}\right]+\left[OH^{-}\right]-\left[H^{+}\right]$$

(5) $$K_{B8.2}=\left[C{O_{3}}^{2-}\right]-\left[CO_{2}\right]+\left[OH^{-}\right]-\left[H^{+}\right]$$

We used 0.1 N HCl and 0.1 N NaOH as titrant according to the following equations:

(6) $$K_{B8.2}=\;\frac{\left(c\;\left(\text{NaOH}\right)\frac{mmol}{l}\right)\left(1000\right)\left(V1\;ml\right)}{V2\;\text{ml}}$$

(7) $$K_{S4.3}=\;\frac{\left(c\;\left(\text{HCl}\right)\frac{\mathrm{mmol}}{l}\right)\left(1000\right)\left(V1\;\mathrm{ml}\right)}{V2\;\text{ml}}$$

where *c* is the molar concentration of titrant, V_1_ the required volume needed to adjust to pH 8.2 or pH 4.3 and V_2_ is the volume of the original sample. For higher accuracy we weighed the sample and converted mass to volume using the density of 1 g ml^−1^. Results are given in mmol l^−1^ or mol/m³ with up to three decimal spaces. TCO_2_ was then calculated according to the formula:

(8) $$\text{TC}O_{2}\left(\text{mg}\;l^{-1}\right)=\left(K_{B8.2}+K_{S4.3}\left(\text{mmol}\;l^{-1}\right)\right)*\left(CO_{2}\;\text{mg}\;\text{mmo}l^{-1}\right)$$

Similarly, we determined total alkalinity (TA) with 0.1 N HCl for acid capacity (K_S4.3_) via endpoint–titration as described above. The concentration of total alkalinity (TA) was then calculated according to the formula:

(9) $$\text{TA}\left(\text{mg}\;l^{-1}\right)=\left(K_{S4.3}\;\text{mmol}\;l^{-1}*\;0.5\right)*\left(\text{CaC}O_{3}\;\text{mg}\;\text{mmo}l^{-1}\right)$$

An example of a parameter scheme, analysis results and PHREECQ keywords of a laboratory water sample (M4-medium) is given in the supplement (Table S1). All parameters were calculated according to the composition of modified M4-medium (Table 3) and to the determination-results of acid- (K_S4.3_) and base capacity (K_B8.2_) (Table 4). These parameters were entered via the listed keywords according to the database-sheet in PHREEQC (Tables S1; S7).

### **Long-term monitoring data**

We analyzed *p*CO_2_ over time from data of four freshwater reservoirs in North-Rhine Westphalia. The reservoirs (Henne, Lister, Möhne and Sorpe) are impoundments of approximately 40 m depth and of an age of 61 to 104 years. Long-term monitoring data of hydrogeochemical- and -physical parameters have been monthly recorded since 1970 and data originate from lake surveys (with accredited laboratory for physicochemical water analyses) by the Ruhr Association in Essen (Ruhrverband). All reservoirs are located in the catchment area of the river Ruhr in the western part of Germany and are fed by different tributaries. Further, the area consists of more than 50–60% forest, only about 0.1% moor and about 2–3% are building area. Geographically, the area consists mainly of sandstones, greywacke and claystone (see supplementary information for details).

The conducted *p*CO_2_ analysis based on PHREEQC uses annual averages of the total inorganic carbon (TIC) converted as total CO_2_ concentration (TCO_2_), as well as the annual averages of pH and temperature in combination with hydrogeochemical and -physical parameters from the years 2016–2018 (Table S5).

We determined TCO_2_ using the total inorganic carbon (TIC; mg l^−1^) and converted to TCO_2_ according to the formula:

(10) $$\text{TC}O_{2}\left(\text{mg}\;l^{-1}\right)=C\left(\text{mmol}\;l^{-1}\right)*M\;\left(CO_{2}\right)\left(\text{mg}\;\text{mmo}l^{-1}\right)$$

(11) $$\text{with}\;C\left(\text{mmol}\;l^{-1}\right)=\frac{C\;\mathrm{anorg}.\;\;\left(\mathrm{TIC}\right)\;\left[\mathrm{mg}\;l^{-1}\right]}{M\;\left(C\right)\;\left[\text{mg}\;{\text{mmol}}^{-1}\right]}$$

## **Results of method validation**

### **Laboratory data**

With regard to the laboratory data, PHREEQC (+) calculations (= ion-balance error ≤5%) resulted in a low discrepancy when calculating *p*CO_2_ based on TA or TCO_2_ (Table 5; Tables S2; S3). Hence, both dissociation constants of carbonic acid (TA and TCO_2_), as well as calculations (PHREEQC (+)) or modelling (PHREEQC (-) = ion-balance error ≥ 99%), provides similar *p*CO_2_–values (Table 5; Tables S2; S3).

**Table 5:** *p*CO_2_ [µatm] results of the different PHREEQC–analyses. TA = PHREEQC-analyses based on total alkalinity determined by acid capacity (K_S4.3_) via endpoint–titration; TCO_2_ = PHREEQC-analyses based on total CO_2_ concentration determined by acid- (K_S4.3_) and base capacity (K_B8.2_) via endpoint-titration. (+) = PHREEQC-analyses with detailed hydrogeochemical and -physical parameters (see Tables 2–4) and an ion-balance error (%) ≤5%; (–) = PHREEQC-analyses only with the parameters pH, TCO_2_ or TA, temperature, as well as density and an ion-balance error (%) ≥99%.

	*p*CO_2_ [µatm]			
pH	TCO_2_ (+)	TCO_2_ (–)	TA (+)	TA (–)
6.369	26915.35	27542.29	22908.68	24547.09
6.386	25118.86	26302.68	24547.09	26915.35
6.406	21877.62	26302.68	20417.38	22387.21
Mean 6.39 ± 0.019 (± SD)	24637.28	26715.88	22624.38	24616.55
6.689	13489.63	14454.40	10964.78	12022.64
6.696	12589.25	13489.63	10715.19	11748.98
6.699	11481.54	12302.69	10715.19	11748.98
Mean 6.70 ± 0.005 (± SD)	12520.14	13415.57	10798.39	11840.20
6.984	5888.44	6309.57	5495.41	6025.60
7.014	5754.40	6165.95	5128.61	5754.40
7.036	5248.07	5623.41	5011.87	5495.41
Mean 7.01 ± 0.026 (± SD)	5630.30	6032.98	5211.96	5758.47
8.075	436.52	478.63	426.58	478.63
8.087	416.87	457.09	407.38	467.74
8.090	416.87	457.09	416.87	467.74
Mean 8.08 ± 0.008 (± SD)	423.42	464.27	416.94	471.37

### **Long-term monitoring data**

The analysis of long-term monitoring data showed comparable results as the laboratory dataset. We found only 3–5% lower *p*CO_2_ levels based on *p*CO_2_-calculations via *p*CO_2_[TCO_2_ (+)] compared to modelled *p*CO_2_ based on *p*CO_2_[TCO_2_ (-)] in all four reservoirs across all successive years (2016–2018) (Table 6; Table S4).

**Table 6:** *p*CO_2_ [µatm] results of different PHREEQC-analyses of four freshwater reservoirs in North-Rhine Westphalia of a long-term monitoring dataset (2016 – 2018). TCO_2_ = PHREEQC-analyses with total CO_2_ concentration determined by the total inorganic carbon (TIC (mg l^−1^)) and calculated as total CO_2_ concentration (TCO_2_ in mg l^−1^) = C (mmol l^−1^) * M (CO_2_) (mg mmol^−1^) with C (mmol l^−1^) = (C anorg. [TIC] (mg l^−1^)) / (M (C) (mg mmol^−1^)). (+) = PHREEQC-analyses with detailed hydrogeochemical and -physical parameters (see Tables S5; S6; e.g. Table 2) and an ion-balance error (%) ≤5%; (–) = PHREEQC-analyses only with the parameters pH, TCO_2_, temperature, as well as density and an ion-balance error (%) ≥99%.

Reservoir	Year	pCO_2_[TCO_2_ (+)][µatm]	pCO_2_[TCO_2_ (-)][µatm]
**Henne**	2016	891.25	912.01
	2017	1000.00	1023.29
	2018	933.25	977.24
**Lister**	2016	1348.96	1380.38
	2017	1862.09	1949.84
	2018	1230.27	1258.93
**Möhne**	2016	1412.54	1479.11
	2017	1122.02	1174.90
	2018	1584.89	1659.59
**Sorpe**	2016	1000.00	1047.13
	2017	1174.90	1202.26
	2018	1122.02	1174.90

**4. POTENTIAL SOURCES OF ERROR**

For a given amount of CO_2_ in solution, the *p*CO_2_ will change significantly with temperature [9]. Indirect methods using only pH, temperature and TA to calculate *p*CO_2_ are manifold but are highly dependent on the quality of measured parameters [1,16]. Small analytical errors may result in large uncertainties in the indirectly calculated TA [1]. In contrast, the quality assessment of a water sample and the laboratory analysis with PHREEQC will help to identify analytic errors of measured parameters.

Especially, in inland waters the choice of TA or TCO_2_ used as an input parameter should depend on the respective water chemistry and buffering capacity of the respective impoundment. Therefore, the use of TCO_2_ as an input parameter for the dissociation constants of carbonic acid is recommended [1,^7^,16], if data collection or measurement techniques provide TIC/DIC or K_B8.2_ in combination with K_S4.3_ (in older datasets also be given as an m- and p-value). These parameters are independent of non-carbonate alkalinity (NCA). Especially in acid, poor buffered inland waters, in which the NCA fraction represents an unknown or even dominant part of alkalinity, it is recommended that estimates of *p*CO_2_ are based on DIC and pH or even direct measurements [1,^7^,16]. Here, it is especially important to avoid degassing as otherwise TIC is reduced and CO_2_ underestimated [28]. If the type and concentration of the NCA is known, it can be accounted for in the calculation of CO_2_ by determining the carbonate contribution as the difference between the total and the non-carbonate alkalinity [28]. Hence, in well-buffered or not acidic and organic rich inland waters and if the NCA fraction is known, also TA is a suitable input parameter. However, input parameters like pH/TA/DIC and water temperature are routinely measured in comprehensive monitoring assays by many environmental agencies [1]. Therefore, these data are readily available to calculate long-term *p*CO_2_-values helping to detect *p*CO_2_ development processes in the past.

**5. FINAL REMARKS**

Here PHREEQC (+) is recommended as a calculation method for the standardized analysis of *p*CO_2_ levels giving realistic *p*CO_2_-calculations of current and past field-, laboratory- and especially long-term data. However, in many long-term datasets there are only a few or even no additional parameters that only allows a modelling of *p*CO_2_ and will result in analyses with an ion-balance error of ≥ 99%. Nevertheless, we here find, that modelled *p*CO_2_ can still be used as an estimation close to the actual *p*CO_2_ level.

Each freshwater body is unique, due to chemical compositions of the underlying geology, climate regimes and/or freshwater chemistry and therefore the *p*CO_2_ and pH relationship is less straight forward compared to the marine system, rendering analyses more difficult. For a global comprehension of freshwater *p*CO_2_, future field and laboratory experiments are needed to further describe *p*CO_2_ dependent freshwater acidification also for different geographic locations. Foremost, this requires a standardized methodology with which current and past *p*CO_2_ can be determined. In future research PHREEQC could be used to analyze the development of current *p*CO_2_ in freshwater from different sources like rivers, lakes and reservoirs capitalizing on long term dataset from global databases (e.g. GloRiCh, Global Reservoir and Dam Database (GRanD), Waterbase (EEA's databases), waterdatabase) or environmental agencies. Further, the advantage of PHREEQC is that with just one method *p*CO_2_ in very different sample types can be analyzed. Here, PHREEQC can help to provide a deeper understanding of *p*CO_2_ regulating and influencing processes predicting future effects of climate change.

## Declaration of Competing Interest

The authors declare that they have no known competing financial interests or personal relationships that could have appeared to influence the work reported in this paper.

## Appendix Supplementary materials

Image, application 1
