# Supplementary material for: Determining freshwater pCO2 based on geochemical calculation and modelling using PHREEQC
Source: MethodsX. 2021 Jun 25;8:101430. doi: 10.1016/j.mex.2021.101430 (PMC8374637; doi:10.1016/j.mex.2021.101430)
Supplement: Supplementary file 1 [file mmc1.docx]

**Supplementary material and/or Additional information**

**Supplementary methods**

***Calculation of mean differences (∆) and mean percentages of pCO_2_ (%)***

To compare *p*CO_2_ results of the respective calculated (+) and modelled (-) PHREEQC-analysis strategies, the mean *p*CO_2_ differences (**∆**) and mean percentages of *p*CO_2_ (%) were calculated.

**∆1** = *p*CO_2_ (x) – *p*CO_2_ (y) and % = 100 x (**∆1** / *p*CO_2_ (y)) (12)

**∆2** = *p*CO_2_ (y) – *p*CO_2_ (x) and % = 100 x (**∆2** / *p*CO_2_ (x)) (13)

where x and y stand for the respective *p*CO_2_–analysis.

**Supplementary tables**

***Tables***

| **Parameters** | **Units** | **PHREEQC–**  **Keyword** | **Input–Parameter** |  |
| --- | --- | --- | --- | --- |
|  |  | Title | M4-medium pH 6.40 |  |
|  |  | Solution | 1 |  |
|  |  | pe | 4.0 |  |
|  |  | units | mg/l |  |
|  |  |  |  |  |
| Temperature | °C | temp | 22.10 |  |
| pH–value |  | pH | 6.406 |  |
| Density | mg kg^-1^ | density | 1.00 |  |
| total CO_2_ concentration (TCO_2_) | mg l^-1^ | C(4)  # or  C | 78.66 as CO2  21.45 as C |  |
| Alkalinity (TA) | mg l^-1^ | Alkalinity | 46.34 |  |
| Calcium | mg l^-1^ | Ca | 80.09 |  |
| Chloride | mg l^-1^ | Cl | 144.46 |  |
| Magnesium | mg l^-1^ | Mg | 12.16 |  |
| Sulphate | mg l^-1^ | S(6) | 48.06 | |
| Potassium | mg l^-1^ | K | 3.144 | |
| Sodium | mg l^-1^ | Na | 18.62 | |
| Oxygen | mg l^-1^ | O(0) | 37.02 | |
| Silicium | mg l^-1^ | Si | 0.494 | |
| Nitrate | mg l^-1^ | N(+5) | 0.199 | |
| Phosphor | mg l^-1^ | P | 0.150 | |
|  |  | END |  | |

**Tab. S1:** Input-example of a laboratory water sample (M4-medium) using keywords. All parameters are based on the composition of M4-medium (Tab. 3; Tab. 4) using default pe-value (pe = 4), equating to 230 mV at the given temperature. Total CO_2_ given by C (4) 78.66 as CO2 (C (4) 78.66 mg l^-1^ = K_S4.3_ + K_B8.2_ (mmol l^-1^) * 44 mg mmol^-1^). K_S4.3_ and K_B8.2_ were determined using endpoint titration (K_B8.2_ by DIN 38409–H7–4–1 and K_S4.3_ by DIN 38409–H7–2; (German Institute for Standardization 2005). Total alkalinity (TA) was analyzed by automated electro-titration with 0.1 N HCl determining the acid capacity (K_S4.3_) via an endpoint-titration. The described alternatives for *p*CO_2_ determination via alkalinity Alkalinity 46.341 (Alkalinity 46.341 mg l^-1^ = (K_S4.3_ (mmol l^-1^) *100,089 mg mmol^-1^ (M CaCO_3_)) / 2) # or to identify the CO_2_ as carbon C 21.454 as C (C 21.454 mg l^-1^ = (K_B8.2_ + K_S4.3_ (mmol l^-1^)) * 12 mg mmol^-1^) (Wisotzky et al. 2018).

|  | *y* (column) | **TA (+)** | | **TCO_2_ (+)** | |  |
| --- | --- | --- | --- | --- | --- | --- |
|  | *x* (row) | ∆ | % | ∆ | % | ∆ |
| **pH 6.39**  ± 0.019  (± SD) | TA (+) | – | – | -2012.89 | -7.95 | -10386.81 |
|  | TCO_2_ (+) | 2012.89 | 8.99 | – | – | -8373.91 |
|  | TA (–) | 1992.17 | 8.82 | -20.73 | 0.23 | -8394.64 |
|  | TCO_2_ (–) | 4091.50 | 18.73 | 2078.61 | 9.09 | -6295.31 |
| **pH 6.70**  ± 0.005  (± SD) | TA (+) | – | – | -1721.75 | -13.43 | -9546.64 |
|  | TCO_2_ (+) | 1721.75 | 15.89 | – | – | -7824.89 |
|  | TA (–) | 1041.81 | 9.65 | -679.94 | -5.07 | -8504.83 |
|  | TCO_2_ (–) | 2617.18 | 24.18 | 895.43 | 7.15 | -6929.45 |
| **pH 7.01**  ± 0.026  (± SD) | TA (+) | – | – | -418.34 | -7.35 | -3693.98 |
|  | TCO_2_ (+) | 418.34 | 8.02 | – | – | -3275.64 |
|  | TA (–) | 546.50 | 10.50 | 128.16 | 2.35 | -3147.48 |
|  | TCO_2_ (–) | 821.01 | 15.75 | 402.68 | 7.15 | -2872.97 |
| **pH 8.08**  ± 0.008  (± SD) | TA (+) | – | – | -6.48 | -1.52 | 327.28 |
|  | TCO_2_ (+) | 6.48 | 1.55 | – | – | 333.75 |
|  | TA (–) | 54.42 | 13.07 | 47.95 | 11.35 | 381.70 |
|  | TCO_2_ (–) | 47.33 | 11.35 | 40.85 | 9.65 | 374.60 |

**Tab. S2:** Comparison of *p*CO_2_ results between different *p*CO_2_ PHREEQC-analysis strategies of a laboratory dataset. Mean differences (**∆**) of the *p*CO_2_ and mean percentages of *p*CO_2_ (%). The row displays the *p*CO_2_ determination method to which the calculations refer. TA = PHREEQC-analyses with alkalinity determined by the acid capacity (K_S4.3_) via endpoint-titration; TCO_2_ = PHREEQC-analyses with total CO_2_ concentration determined by the acid- (K_S4.3_) and base capacity (K_B8.2_) measured via endpoint-titration. (+) = PHREEQC-analyses with detailed hydrogeochemical and -physical parameters (see Tab. 2 - 4) and an ion–balance error (%) ≤5%; (–) = PHREEQC-analyses only with the parameters pH, TCO_2_ or TA, temperature, as well as density and an ion-balance error (%) ≥99%.

|  | | **TA (+)**  **–**  **TCO_2_ (+)** | | **TCO_2_ (+)**  **–**  **TCO_2_ (–)** | | **TA (+)**  **–**  **TA (–)** | | **TA (–)**  **–**  **TCO_2_ (–)** | | |  |
| --- | --- | --- | --- | --- | --- | --- | --- | --- | --- | --- | --- |
| pH | Differences | | Mean  %  (*p*CO_2_) | Differences | Mean  %  (*p*CO_2_) | Differences | Mean  %  (*p*CO_2_) | | Differences | Mean  %  (*p*CO_2_) | |
| 8.09 | | -9.49 | -2.28 | -40.22 | -8.80 | -60.35 | -12.90 | | 10.65 | 2.33 | |
| 8.09 | | 0.00 | 0.00 | -40.22 | -8.80 | -50.87 | -10.87 | | 10.65 | 2.33 | |
| 8.08 | | -9.94 | -2.28 | -42.11 | -8.80 | -52.05 | -10.87 | | 0.00 | 0 | |
| 7.01 | | -625.79 | -10.87 | -411.55 | -6.67 | -625.79 | -10.87 | | -411.55 | -6.67 | |
| 6.98 | | -393.03 | -6.67 | -421.14 | -6.67 | -530.19 | -8.80 | | -283.98 | -4.50 | |
| 7.04 | | -236.20 | -4.50 | -375.34 | -6.67 | -483.54 | -8.80 | | -128.00 | -2.28 | |
| 6.70 | | -766.34 | -6.67 | -821.15 | -6.67 | -1033.78 | -8.80 | | -553.71 | -4.50 | |
| 6.70 | | -1874.06 | -14.89 | -900.37 | -6.67 | -1033.78 | -8.80 | | -1740.65 | -12.90 | |
| 6.69 | | -2524.85 | -18.72 | -964.77 | -6.67 | -1057.86 | -8.80 | | -2431.75 | -16.82 | |
| 6.37 | | -4006.67 | -14.89 | -626.94 | -2.28 | -1638.41 | -6.67 | | -2995.20 | -10.87 | |
| 6.39 | | -571.78 | -2.28 | -1183.82 | -4.50 | -2368.26 | -8.80 | | 612.67 | 2.33 | |
| 6.41 | | -1460.24 | -6.67 | -4425.06 | -16.82 | -1969.83 | -8.80 | | -3915.47 | -14.89 | |

**Tab. S3:** Data for the differences and mean (%) calculation comparing the different *p*CO_2_ PHREEQC analysis strategies of laboratory data. Same definition of analysis- and calculation methods as in Tab. S2.

|  |  | **TCO_2_ (+) vs.**  **TCO_2_ (–)** | |
| --- | --- | --- | --- |
|  |  | ∆ | % |
| **Henne** | 2016 | -20.76 | -2.28 |
|  | 2017 | -23.29 | -2.28 |
|  | 2018 | -43.98 | -4.50 |
| **Lister** | 2016 | -31.42 | -2.28 |
|  | 2017 | -87.76 | -4.50 |
|  | 2018 | -28.66 | -2.28 |
| **Möhne** | 2016 | -66.57 | -4.50 |
|  | 2017 | -52.88 | -4.50 |
|  | 2018 | -74.69 | -4.50 |
| **Sorpe** | 2016 | -47.13 | -4.50 |
|  | 2017 | -27.37 | -2.28 |
|  | 2018 | -52.88 | -4.50 |

**Tab. S4:** Mean differences (**∆**) of the *p*CO_2_ and mean percentages of *p*CO_2_ (%) of four freshwater reservoirs in North-Rhine Westphalia of a long-term monitoring dataset (2016 to 2018). TCO_2_ = PHREEQC-analyses with total CO_2_ concentration determined by the total inorganic carbon (TIC (mg l^-1^)) and calculated as total CO_2_ concentration (mg l^-1^) = C in mmol l^-1^* M (CO_2_) mg mmol^-1^ with C (mmol l^-1^) = (C anorg. (TIC) [mg l^-1^]) / (M (C) [mg mmol^-1^]). (+) = PHREEQC-analyses with detailed hydrogeochemical and -physical parameters and an ion-balance error (%) ≤5%; (–) = PHREEQC-analyses only with the parameters pH, TCO_2_ or TA, temperature, as well as density and an ion-balance error (%) ≥99%

|  | **Henne 2016** | **Henne 2017** | **Henne**  **2018** | **Lister 2016** | **Lister 2017** | **Lister 2017** | **Möhne**  **2016** | **Möhne**  **2017** | **Möhne**  **2018** | **Sorpe 2016** | **Sorpe 2017** | **Sorpe 2018** |
| --- | --- | --- | --- | --- | --- | --- | --- | --- | --- | --- | --- | --- |
| **Temp** | 11.52 | 9.01 | 8.03 | 13.31 | 9.55 | 7.74 | 12.75 | 11.29 | 9.21 | 11.41 | 8.74 | 7.29 |
| **pH** | 7.86 | 7.83 | 7.90 | 7.60 | 7.27 | 7.365 | 7.86 | 7.93 | 7.77 | 7.78 | 7.70 | 7.77 |
| **C(4)** | 57.17 | 61.82 | 69.03 | 47.69 | 34.98 | 28.36 | 90.14 | 85.19 | 86.19 | 53.26 | 54.31 | 62.75 |
| **O(0)** | 8.82 | 7.90 | 10.45 | 8.54 | 7.89 | 9.20 | 7.59 | 7.40 | 8.40 | 9.93 | 10.08 | 10.26 |
| **Amm** | 0.03 | 0.04 | 0.04 | 0.02 | 0.023 | 0.05 | 0.036 | 0.059 | 0.05 | 0.02 | 0.02 | 0.03 |
| **Si** |  |  |  | 1.21 | 1.45 | 1.50 |  |  |  | 1.09 | 1.18 | 1.79 |
| **N(+3)** |  |  |  |  |  |  |  |  |  |  |  |  |
| **N(+5)** | 2.98 | 2.86 | 2.71 | 1.61 | 1.74 | 1.62 | 1.3 | 1.59 | 1.71 | 1.73 | 1.61 | 1.77 |
| **Fe(+2)** |  | 0.05 |  |  | 0.04 |  |  | 0.04 |  |  | 0.01 |  |
| **Mn(+2)** |  | 0.06 |  |  | 2.63 |  |  | 0.74 |  |  | 0.0 |  |
| **Na** | 7.40 | 7.70 | 7.65 | 8.85 | 8.23 | 10.20 | 28.00 | 27.67 | 26.00 | 5.70 | 7.00 | 5.90 |
| **K** | 1.25 | 1.33 | 1.20 | 1.00 | 0.97 | 0.98 | 2.40 | 2.67 | 2.40 | 0.75 | 1.25 | 1.04 |
| **Ca** | 29.00 | 32.33 | 33.00 | 13.50 | 13.67 | 14.00 | 37.00 | 37.67 | 37.5 | 15.80 | 20.5 | 26.50 |
| **Mg** | 3.70 | 4.067 | 3.90 | 3.15 | 3.13 | 3.20 | 5.20 | 5.50 | 5.20 | 3.25 | 4.00 | 4.40 |
| **Cl** | 10.71 | 12.25 | 12.50 | 15.86 | 16.86 | 20.50 | 37.00 | 38.75 | 38.00 | 9.81 | 10.88 | 11.00 |
| **S(6)** | 18.14 | 19.00 | 19.00 | 11.71 | 10.71 | 10.50 | 22.00 | 22.25 | 21.5 | 18.14 | 18.38 | 18.00 |

**Tab. S5:** Yearly average of total inorganic carbon (TIC), as well as pH and temperature in combination with hydrogeochemical and physical parameters of the long-term datasets of four freshwater reservoirs in North-Rhine Westphalia. Total inorganic carbon (TIC) converted as total CO_2_ concentration (TCO_2_; here as C (4)). All parameters instead of pH and temperature are in the units mg l^-1^. O(0) = O_2_ concentration; Amm= Ammonium; Si= Silicium; N(+3)= Nitrite; N(+5)= Nitrate; Fe(+2)= Iron; Mn(+2)= Manganese; Na= Sodium; K= Potassium; Ca= Calcium; Mg= Magnesium; Cl= Chloride and S(6)= Sulfate.

| **Application-parameters** | **possible Input-parameters** | **Input combinations** | **Ion-balance**  **error (%)** | **Results** |
| --- | --- | --- | --- | --- |
| **Carbon content** | TIC  Total CO_2_ concentration  Alkalinity | Carbon content  +  Physical parameters  +  Hydrogeo-  chemical parameters | 0 – 25% | - Gas– and minerals phase (Saturation indices):   (CO_2 (g)_, Calcite, Aragonite, etc.)   - Quality evaluation of the water analysis:   Ion-balance error  Ion strength   - Justification of the Input–parameters:   pH, the redox reaction, temperature, etc.  (if necessary)   - Distribution of species of the dissolved aquatic complexes:   HCO_3_^–^ , CaCO_3_, CO_3_^2–^, H^+^, OH^–^, Ca(HCO_3_)_2_, MgHCO_3_^+^,  CaSO_4_, etc. |
| **Physical Parameters** | Temperature  pH  Density |  |  |  |
| **Hydrogeo-chemical and -physical Parameters** | Ionic parameters:  Cation:  (Mg^2+^, CS2^+^, K^+^, Na^+^, Fe, Mn, etc.)  Anion:  (NO_3_^–^, NO_2_^–^, SO_4_^2–^, Cl^–^, etc.) |  |  |  |
|  |  | Carbon content | > 90% | - Gas– and minerals phase (Saturation indices):   (CO_2 (g)_, Calcite, Aragonite, etc.) |
|  |  | Carbon content  +  Physical parameters |  |  |

**Tab. S6:** Application overview of PHREEQC with required parameters, its input-possibilities, -definitions and -combinations. All input-combinations lead to the listed results, but quality of the output, significance and quantity is depended on completeness of the input-parameters and water analysis.

| *------------------*  *Reading data base.*  *------------------*  *SOLUTION_MASTER_SPECIES*  *SOLUTION_SPECIES*  *PHASES*  *EXCHANGE_MASTER_SPECIES*  *EXCHANGE_SPECIES*  *SURFACE_MASTER_SPECIES*  *SURFACE_SPECIES*  *RATES*  *END*  *------------------------------------*  *Reading input data for simulation 1.*  *------------------------------------*  *Title M4 Medium_pH 6.4 12*  *SOLUTION 1*  *temp 22.100*  *units mg/l*  *density 1.000*  *pe 4.0*  *pH 6.406*  *Ca 80.09406205*  *Cl 144.4606305*  *Mg 12.15892954*  *S(6) 48.05591156*  *K 3.144938846*  *Na 18.61695962*  *Si 0.494130463*  *N(+5) 0.199885827*  *P 0.150226111*  *H(0) 0.779592135*  *O(0) 37.02365667*  *C(4) 78.66385283 as CO2*  *END*  *-----*  *TITLE*  *-----*  *M4 Medium_pH 6.4 12*  *-------------------------------------------*  *Beginning of initial solution calculations.*  *-------------------------------------------*  *Initial solution 1.*  *-----------------------------Solution composition------------------------------*  *Elements Molality Moles*  *C(4) 1.788e-03 1.788e-03*  *Ca 1.999e-03 1.999e-03*  *Cl 4.076e-03 4.076e-03*  *H(0) 7.737e-04 7.737e-04*  *K 8.046e-05 8.046e-05*  *Mg 5.003e-04 5.003e-04*  *N(5) 1.428e-05 1.428e-05*  *Na 8.101e-04 8.101e-04*  *O(0) 2.315e-03 2.315e-03*  *P 4.852e-06 4.852e-06*  *S(6) 5.005e-04 5.005e-04*  *Si 8.227e-06 8.227e-06*  *----------------------------Description of solution----------------------------*  *pH = 6.406*  *pe = 4.000*  *Specific Conductance (uS/cm, 22 oC) = 628*  *Density (g/cm3) = 0.99808 (Millero)*  *Activity of water = 1.000*  *Ionic strength = 8.577e-03*  *Mass of water (kg) = 1.000e+00*  *Total alkalinity (eq/kg) = 9.850e-04*  *Total CO2 (mol/kg) = 1.788e-03*  *Temperature (deg C) = 22.100*  *Electrical balance (eq) = -1.918e-04*  *Percent error, 100*(Cat-\|An\|)/(Cat+\|An\|) = -1.66*  *Iterations = 10*  *Total H = 1.110142e+02*  *Total O = 5.551519e+01*  *---------------------------------Redox couples---------------------------------*  *Redox couple pe Eh (volts)*  *H(0)/H(1) -6.2737 -0.3675*  *O(-2)/O(0) 14.6227 0.8566*  *----------------------------Distribution of species----------------------------*  *Log Log Log*  *Species Molality Activity Molality Activity Gamma*  *H+ 4.274e-07 3.926e-07 -6.369 -6.406 -0.037*  *OH- 2.249e-08 2.040e-08 -7.648 -7.690 -0.042*  *H2O 5.551e+01 9.998e-01 1.744 -0.000 0.000*  *C(4) 1.788e-03*  *HCO3- 9.639e-04 8.785e-04 -3.016 -3.056 -0.040*  *CO2 8.043e-04 8.059e-04 -3.095 -3.094 0.001*  *CaHCO3+ 1.546e-05 1.409e-05 -4.811 -4.851 -0.040*  *MgHCO3+ 3.691e-06 3.354e-06 -5.433 -5.474 -0.042*  *NaHCO3 3.626e-07 3.633e-07 -6.441 -6.440 0.001*  *CaCO3 2.064e-07 2.068e-07 -6.685 -6.684 0.001*  *CO3-2 1.432e-07 9.874e-08 -6.844 -7.006 -0.161*  *MgCO3 2.970e-08 2.976e-08 -7.527 -7.526 0.001*  *NaCO3- 1.284e-09 1.167e-09 -8.891 -8.933 -0.042*  *Ca 1.999e-03*  *Ca+2 1.912e-03 1.318e-03 -2.719 -2.880 -0.162*  *CaSO4 7.146e-05 7.160e-05 -4.146 -4.145 0.001*  *CaHCO3+ 1.546e-05 1.409e-05 -4.811 -4.851 -0.040*  *CaHPO4 3.394e-07 3.401e-07 -6.469 -6.468 0.001*  *CaCO3 2.064e-07 2.068e-07 -6.685 -6.684 0.001*  *CaH2PO4+ 1.121e-07 1.019e-07 -6.950 -6.992 -0.042*  *CaPO4- 2.134e-09 1.939e-09 -8.671 -8.712 -0.042*  *CaOH+ 6.129e-10 5.568e-10 -9.213 -9.254 -0.042*  *CaHSO4+ 1.750e-10 1.590e-10 -9.757 -9.799 -0.042*  *Cl 4.076e-03*  *Cl- 4.076e-03 3.698e-03 -2.390 -2.432 -0.042*  *H(0) 7.737e-04*  *H2 3.869e-04 3.876e-04 -3.412 -3.412 0.001*  *K 8.046e-05*  *K+ 8.031e-05 7.286e-05 -4.095 -4.138 -0.042*  *KSO4- 1.498e-07 1.361e-07 -6.824 -6.866 -0.042*  *KHPO4- 7.774e-11 7.063e-11 -10.109 -10.151 -0.042*  *KOH 6.420e-13 6.433e-13 -12.192 -12.192 0.001*  *Mg 5.003e-04*  *Mg+2 4.764e-04 3.302e-04 -3.322 -3.481 -0.159*  *MgSO4 2.005e-05 2.009e-05 -4.698 -4.697 0.001*  *MgHCO3+ 3.691e-06 3.354e-06 -5.433 -5.474 -0.042*  *MgHPO4 1.150e-07 1.152e-07 -6.939 -6.938 0.001*  *MgH2PO4+ 3.578e-08 3.251e-08 -7.446 -7.488 -0.042*  *MgCO3 2.970e-08 2.976e-08 -7.527 -7.526 0.001*  *MgOH+ 2.579e-09 2.344e-09 -8.588 -8.630 -0.042*  *MgPO4- 7.215e-10 6.555e-10 -9.142 -9.183 -0.042*  *N(5) 1.428e-05*  *NO3- 1.428e-05 1.293e-05 -4.845 -4.888 -0.043*  *Na 8.101e-04*  *Na+ 8.087e-04 7.354e-04 -3.092 -3.133 -0.041*  *NaSO4- 1.115e-06 1.013e-06 -5.953 -5.995 -0.042*  *NaHCO3 3.626e-07 3.633e-07 -6.441 -6.440 0.001*  *NaCO3- 1.284e-09 1.167e-09 -8.891 -8.933 -0.042*  *NaHPO4- 7.847e-10 7.130e-10 -9.105 -9.147 -0.042*  *NaOH 1.235e-11 1.237e-11 -10.908 -10.908 0.001*  *O(0) 2.315e-03*  *O2 1.157e-03 1.160e-03 -2.936 -2.936 0.001*  *P 4.852e-06*  *H2PO4- 3.515e-06 3.196e-06 -5.454 -5.495 -0.041*  *HPO4-2 7.310e-07 4.972e-07 -6.136 -6.303 -0.167*  *CaHPO4 3.394e-07 3.401e-07 -6.469 -6.468 0.001*  *MgHPO4 1.150e-07 1.152e-07 -6.939 -6.938 0.001*  *CaH2PO4+ 1.121e-07 1.019e-07 -6.950 -6.992 -0.042*  *MgH2PO4+ 3.578e-08 3.251e-08 -7.446 -7.488 -0.042*  *CaPO4- 2.134e-09 1.939e-09 -8.671 -8.712 -0.042*  *NaHPO4- 7.847e-10 7.130e-10 -9.105 -9.147 -0.042*  *MgPO4- 7.215e-10 6.555e-10 -9.142 -9.183 -0.042*  *KHPO4- 7.774e-11 7.063e-11 -10.109 -10.151 -0.042*  *PO4-3 1.282e-12 5.384e-13 -11.892 -12.269 -0.377*  *S(6) 5.005e-04*  *SO4-2 4.077e-04 2.799e-04 -3.390 -3.553 -0.163*  *CaSO4 7.146e-05 7.160e-05 -4.146 -4.145 0.001*  *MgSO4 2.005e-05 2.009e-05 -4.698 -4.697 0.001*  *NaSO4- 1.115e-06 1.013e-06 -5.953 -5.995 -0.042*  *KSO4- 1.498e-07 1.361e-07 -6.824 -6.866 -0.042*  *HSO4- 1.105e-08 1.004e-08 -7.957 -7.998 -0.042*  *CaHSO4+ 1.750e-10 1.590e-10 -9.757 -9.799 -0.042*  *Si 8.227e-06*  *H4SiO4 8.224e-06 8.241e-06 -5.085 -5.084 0.001*  *H3SiO4- 3.073e-09 2.792e-09 -8.512 -8.554 -0.042*  *H2SiO4-2 5.859e-16 3.992e-16 -15.232 -15.399 -0.167*  *------------------------------Saturation indices-------------------------------*  *Phase SI log IAP log KT*  *Anhydrite -2.08 -6.43 -4.35 CaSO4*  *Aragonite -1.57 -9.89 -8.32 CaCO3*  *Calcite -1.42 -9.89 -8.46 CaCO3*  *Chalcedony -1.50 -5.08 -3.59 SiO2*  *Chrysotile -14.74 17.82 32.56 Mg3Si2O5(OH)4*  *CO2(g) -1.66 -3.09 -1.43 CO2*  *Dolomite -3.35 -20.37 -17.02 CaMg(CO3)2*  *Gypsum -1.85 -6.43 -4.58 CaSO4:2H2O*  *H2(g) -0.27 -3.41 -3.14 H2*  *H2O(g) -1.59 -0.00 1.59 H2O*  *Halite -7.14 -5.57 1.58 NaCl*  *Hydroxyapatite -4.53 -7.69 -3.16 Ca5(PO4)3OH*  *O2(g) -0.06 -2.94 -2.87 O2*  *Quartz -1.08 -5.08 -4.00 SiO2*  *Sepiolite -12.43 3.41 15.84 Mg2Si3O7.5OH:3H2O*  *Sepiolite(d) -15.25 3.41 18.66 Mg2Si3O7.5OH:3H2O*  *SiO2(a) -2.35 -5.08 -2.74 SiO2*  *Talc -14.08 7.66 21.73 Mg3Si4O10(OH)2*  *------------------*  *End of simulation.*  *------------------*  *------------------------------------*  *Reading input data for simulation 2.*  *------------------------------------*  *-----------*  *End of run.*  *-----------* |
| --- |

**Tab. S7:** PHREEQC-Output sheet of a water sample from the laboratory experiment for the *p*CO_2_ -conditions of pH 6.406 with the analysis results of Tab. 2 - 4. The above described analysis of *p*CO_2_ based on the direct measurement of the dissociation constants, here i.e. total CO_2_ concentration (TCO_2_) entering as "C (4) 78.664 as CO2" into the PHREEQC-Input sheet together with hydrogeochemical and -physical parameters of Tab. 2 - 4.

**Additional information**

**Monitoring data**

Monthly long-term monitoring data was recorded by the Ruhr Association in Essen.

Measurements of physicochemical data of nearly all water depths over the last 40 years were provided. Data from the years 1974 to 1998 were digitized and stored in Excel (Microsoft Office, 2018). Data from 1999 to 2018 was provided as digital records.

Möhne reservoir

The Möhne reservoir is located close to the city Soest in North Rhine-Westphalia. The dammed rivers Möhne and Heve provide a mean annual inflow rate of 192.6 million m^3^ (1961-2005) and form a surface area of 10.67 km^2^. Where Möhne and Heve flow in the reservoir, the Ruhr Association build for each river a pre-reservoir with a constant water level. This ensures a reduced entry of sediments and nutrients in the main reservoir. The 650 m long and up to 40 m high mansard dam ensures a storage capacity of 134.5 million m^3^ at maximum storage level of 213.74 m above mean sea level. This leads to an intermediate depth of 12.61 m and a maximum depth of 33.7 m. The Möhne reservoir covers nearly 25 % of the storage capacity of all reservoirs along the Ruhr. The catchment area of 436.4 km^2^ consists of more than 50 % forest only about 3 % are building area.

Lister reservoir

The Lister reservoir was commissioned in 1912 and has been operationally integrated operationally in the Bigge reservoir in 1965. It became one of the five pre-reservoirs. The difference in water level between Lister and Bigge reservoir is used for power generation. The Lister reservoir is located in the south of Sauerland, near the city Attendorn. The dammed rivers Lister, Beche and the Herpeler creek provide a mean annual inflow rate of 66.5 million m^3^ and form a surface area of 1.68 km^2^. The Lister dam ensures a storage capacity of 21.6 million m^3^ at maximum storage level of 319.45 m above mean sea level. This leads to an intermediate depth of 12.86 m and a maximum depth of 35.6 m. The catchment area of 67.9 km^2^ consists of more than 50 % forest, only about 0.1 % moor and about 2 % are building area.

Sorpe Reservoir

In 1935, the Sorpe reservoir was commissioned. The reservoir was built as so-called carry-over storage. This means that the storage capacity is greater than the mean annual inflow, i.e. a reservoir that needs more than one year to fill up. The Sorpe reservoir is located in the north of Sundern. The dammed river Sorpe is a tributary of the river Röhr and provides a mean annual inflow rate of 44 million m^3^ (1961-1993) and forms a surface area of 3.38 km^2^. The pre-reservoir of the Sorpe reservoir is separated by the pre-dam Amecke from the main basin, which leads to a constant water level, irrespective of the fluctuations in the main pool. With a height of 69 m above the foundation bed, the Sorpe dam (earthfill dam with concrete core wall) was in turn the highest earth dam in Germany. The Sorpe dam has a storage capacity of 70 million m^2^ at maximum storage level of 283.03 m above mean sea level. This makes an intermediate depth of 20.83 m and a maximum depth of 57 m. The catchment area of 97.7 km^2^ consists of more than 60 % forest, only about 3 % are building area. The data used in this study were collected and documented in a depth profile every month about 100 m in front of the dam.

Henne Reservoir

The Henne dam and reservoir was built on the Henne river between 1901 and 1905. This masonry dam with a height of 38 metres formed an impounded of eleven million cubic metres of water. This dam was decom-missioned in 1949 and replaced by the new Henne dam and reservoir with a storage capacity of 38.4 million m^3^ in 1950-1955. The Henne reservoir serves to ensure water supply, flood protection and hydropower generation and is used for recreational purposes. The main reservoir has a maximum depth of 30 metres.
